# Latent profiles of resilience and associations with quality of life in head and neck cancer patients undergoing proton and heavy ion therapy

**DOI:** 10.3389/fonc.2023.1270870

**Published:** 2024-01-17

**Authors:** Lina Xiang, Hongwei Wan, Yu Zhu, Shuman Wang, Mimi Zheng

**Affiliations:** ^1^Department of Nursing, Shanghai Proton and Heavy Ion Center, Fudan University Cancer Hospital, Shanghai, China; ^2^Shanghai Key Laboratory of Radiation Oncology (20dz2261000), Shanghai, China; ^3^Shanghai Engineering Research Center of Proton and Heavy Ion Radiation Therapy, Shanghai, China

**Keywords:** head and neck cancer, resilience, quality of life, latent profile analysis, cancer

## Abstract

**Background:**

Psychological resilience is the most important psychological protection factor for cancer patients in the face of tumors and treatment. However, few studies have performed meaningful latent profile analyses of resilience to identify unobserved subgroups of head and neck cancer patients.

**Purpose:**

The purpose of this study was to investigate the characteristics of resilience in head and neck cancer patients using latent profile analysis (LPA) to determine the sociodemographic and disease characteristics of each profile. In particular, we examined the association of different resilience profiles with the quality of life of head and neck cancer patients.

**Methods:**

A total of 254 head and neck cancer patients completed a demographic questionnaire, the Resilience Scale Specific to Cancer and the EOTRC QLQ-C3O, used to assess their resilience and quality of life.

**Results:**

LPA identified three distinct profiles based on varying levels of resilience: “low resilience” group (*n* = 45; 17.72%), “moderate resilience” group (*n* = 113; 44.49%), and “high resilience” group (*n* = 96; 37.80%). Gender (*χ*^2^ = 6.20; *p* < 0.01), education level (*χ*^2^ = 1,812.59; *p* < 0.01), treatment regimen (*χ*^2^ = 6.32; *p* < 0.01), tumor stage (*χ*^2^ = 3.92; *p* ≤ 0.05), and initial recurrence (*χ*^2^ = 5.13; *p* < 0.05) were important predictors. High resilience was significantly related to higher quality of life (*χ*^2^ = 15.694; *p* < 0.001).

**Conclusions:**

Head and neck cancer patients’ psychological resilience can be categorized as three resilience profiles; those who are female and have a low education level tend to have lower psychological resilience. Low resilience in patients is linked to poor role function and social function, low quality of life, and more severe pain symptoms, highlighting the need to address resilience in patient care for improved wellbeing.

## 1 Introduction

Head and neck cancer (HNC) is a prevalent and challenging disease, ranking as the sixth most common tumor worldwide (1), and can involve the nasopharynx, oropharynx, hypopharynx, larynx, oral cavity, paranasal sinuses and nasal cavity, salivary glands, and thyroid gland (2). Its high morbidity, recurrence, and mortality rates pose significant biopsychosocial challenges to patients, leading to a decreased quality of life (3). HNC patients undergoing proton and heavy ion therapy experience a diminished quality of life due to the physical symptoms, psychological impact, and social and functional implications associated with the treatment (4). HNC patients experience serious psychological distress, which significantly impacts their quality of life (5). A survey of 3.59 million American cancer patients found that the suicide rate among HNC patients was the highest among all cancer populations (6); the suicide rate among HNC patients is four times that of the general population (7). Therefore, it is necessary to pay attention to the psychological distress of HNC patients.

Psychological resilience can improve quality of life by reducing psychological distress among cancer patients. Psychological resilience is the ability to adapt and recover from adversity and stress (8). A few studies have shown that a subset of HNC patients exhibit higher levels of psychological resilience, enabling them to adapt and cope effectively with the emotional and physical burdens of their illness (9). However, it is important to note that not all HNC patients exhibit high levels of resilience (10). Understanding the various factors that contribute to resilience, as well as identifying those patients who may be at risk of low resilience, is essential for developing targeted interventions and support programs to enhance the psychological wellbeing of HNC patients.

Factors contributing to resilience in cancer patients have been explored, including social support networks, coping strategies, and individual characteristics such as optimism and self-efficacy (11). However, there is limited literature that utilizes latent profile analysis (LPA) to explore the factors influencing psychological resilience in HNC patients. LPA is a statistical method used to uncover the underlying group structures in data by dividing individuals into different groups or profiles, revealing the latent structures and differences among groups in the data (12). By employing LPA, researchers can identify distinct profiles or subgroups of HNC patients based on their resilience levels and associated factors. This approach provides a more comprehensive understanding of the complex interplay between resilience and various influencing factors. It is worth noting that the application of LPA in studying psychological resilience in HNC patients is still relatively underutilized.

Furthermore, the relationship between psychological resilience and quality of life in HNC patients warrants further exploration. MacDonald’s study described the levels of psychological resilience and examined its relationship to quality of life in HNC patients. However, it did not explore this relationship within specific subgroups (10). Applying LPA in studying HNC patients, we can gain insights into the various patterns of psychological resilience and understand how these patterns relate to their overall quality of life, helping researchers and practitioners tailor interventions and support strategies to meet the unique needs of each subgroup.

Therefore, this research aimed to (1) investigate psychological resilience latent profiles of HNC patients; (2) explore the sociodemographic and disease characteristics of each profile; and (3) compare quality of life and its various dimensions in each resilience profile, providing references for targeted and individualized interventions for HNC patients.

## 2 Method

### 2.1 Participants

We used a descriptive correlational study designed to recruit HNC patients who received proton and heavy ion radiotherapy in Shanghai from January to September 2021. The eligibility criteria were as follows: ① an age older than 18 years; ② a clinical diagnosis of HNC; ③ an education level of elementary school or above and the ability to understand and answer questions correctly; and ④ willingness to participate in the questionnaire survey. The exclusion criteria were as follows: ① individuals with severe cardiac, pulmonary, renal failure, and tumor metastasis; ② individuals with cognitive dysfunction or mental illness; ③ individuals receiving relevant psychotherapy during treatment; and ④ individuals who did not know their disease condition.

### 2.2 Sample size calculation

According to the statistical requirements of multifactorial analysis, the sample size of a study is usually 5 to 10 times the number of the independent variables, and this study included 8 sociodemographic variables (age, gender, marital status, children, education level, employment status, annual income, and insurance status), 6 disease characteristic variables (tumor site, tumor stage, time of diagnosis, history of tumor-related surgeries, initial recurrence, and treatment regimen), and 2 psychological resilience and quality-of-life variables, for a total of 16 variables. Additionally, based on the statistical requirements of LPA, this study requires a minimum of 200 HNC patients as the sample size. Therefore, considering a 20% invalidity rate, a total of 260 questionnaires were distributed in this study.

### 2.3 Instruments

#### 2.3.1 Demographic questionnaire

The general information questionnaire was a self-designed general demographic questionnaire that included age, gender, marital status, children, education level, employment status, annual family income, and health insurance status. The questionnaire also included disease characteristics, such as tumor site, tumor stage, time of diagnosis, history of tumor-related surgeries, initial recurrence, and treatment regimen.

#### 2.3.2 Resilience scale specific to cancer

This scale is the only scale developed to evaluate the psychological resilience of cancer patients (13). It includes 25 items divided into five dimensions: nonspecific resilience components (Items 1–6), disease benefits (Items 7–11), support and response (Items 12–16), hope for the future (Items 17–21), and meaning of existence (Items 22–25). The scale is scored using a five-point Likert scale, and the total score is 25–125 points. The higher the score is, the better the resilience. The Cronbach’s *α* coefficient is 0.85, and the structural validity (CFI) is 0.901, which indicates good reliability and validity.

#### 2.3.3 The European organization for research and treatment of cancer, quality of life questionnaire

This scale is used to evaluate the quality of life of cancer patients (14), and includes 30 items, five functional areas, three symptom areas, and six single items to measure patient symptoms using a four-point Likert scale. Items 29 and 30 are used to assess the overall quality of life of the patient, with a score of 1–7. The Cronbach’s *α* coefficient of the scale is 0.73. To compare the scores of various fields, the rough scores are converted into standardized scores of 0–100 in five functional areas and three symptom areas. For the functional fields and general health fields, higher scores indicate better functional status and quality of life. For the symptom fields, higher scores indicate more symptoms or questions.

### 2.4 Data collection procedure

This study received approval from the hospital ethics committee of REDACTED. Two nursing graduate students collected and distributed the questionnaires in the hospital. The distribution time was 1 week before the patient was discharged, and the questionnaire was completed in a quiet meeting room. The participants were counseled about the aims and other details of our study. This study obtained informed consent from patients prior to their enrollment. Each patient completed this questionnaire in 10–15 min.

### 2.5 Statistical analysis

The questionnaire database was established by EpiData3.1 software, and the data were entered independently by two researchers. After the double check, we used SPSS 24.0 and Mplus 7.0 software to perform the statistical analysis of the data. Descriptive analysis was used to analyze HNC patients’ sociodemographic data, psychological resilience, and quality of life.

LPA was used to examine the latent profiles of psychological resilience. First, the model took a profile and gradually increased the number of model profiles until the optimal model was generated. The model was assessed using the model-related fitting index (15), which consists mostly of the information evaluation index indicators: the Akaike Information criterion (AIC), Bayesian information criterion (BIC), and sample-corrected BIC (aBIC). The smaller the statistical value was, the better the model fit. Entropy was used to measure the model’s accuracy in classifying data. The accuracy was larger than 0.9, meaning that the classification accuracy may exceed 90%. The Lo–Mendell–Rubin corrected likelihood ratio (LMR) and the bootstrap-based likelihood ratio test (BLRT) were used to compare the difference between k profiles and k-1 profiles, and *p* < 0.05 indicated that the K profile had a better fitting effect.

After the optimal model was chosen, the difference in sociodemographic and disease variables in each resilience profile was obtained by using chi‐squared tests. The data collected from the questionnaire consist of qualitative data, which do not follow a normal distribution. As a result, Kruskal–Wallis test was applied to compare quality of life and its dimension in each latent profile. *p*-values <0.05 were considered indicative of statistical significance.

## 3 Results

A total of 260 questionnaires were distributed, and 254 patients were ultimately included, with a loss-to-follow-up rate of 97% (for instance, questionnaires that showed a particular pattern or contradicting responses or had incomplete answers). The lowest score of resilience was 51 points, and the highest score was 123 points, with an average of 97.07 ± 14.02 points. The lowest score of overall quality of life was 16.67 points, and the highest was 100 points, with an average of 81.19 ± 15.90 points.

### 3.1 Characteristics of patients

The demographic and clinical characteristics of the patients and resilience in different categories of variables are described in **Table 1**. The mean age of the patients was 44.07 years (SD =11.89, range 18–79).

**Table 1:** Demographic and disease characteristics of participants (*N* = 254).

Variables	Item	*N*	%
Gender	Female	94	37.0
	Male	160	63.0
Marital status	Unmarried	44	17.3
	Married	210	82.7
Children	No	64	25.2
	Yes	190	74.8
Education level	Middle school or lower	85	33.5
	High school	130	51.2
	College degree or higher	39	15.3
Employment status	Unemployed	125	49.2
	Employed	129	50.8
Annual income (RMB)	<150,000	76	29.9
	150,000–300,000	71	28.0
	>300,000	107	42.1
Insurance status	No	81	31.9
	Yes	173	68.1
Tumor site	Nasopharyngeal	113	44.5
	Oral cavity	42	16.5
	Larynx	28	11.0
	Oropharynx	71	28.0
Tumor stage	I or II	103	40.6
	III or IV	151	59.4
Time of diagnosis	0–3 months	79	31.1
	3–6 months	71	28.0
	>6 months	104	40.9
History of tumor-related surgeries	No	68	26.8
	Yes	186	73.2
Initial recurrence	No	168	66.4
	Yes	85	33.6
Treatment regimen	X-ray + heavy ion	54	21.3
	Protons + heavy ion	62	24.4
	Protons	86	33.9
	Heavy ion	52	20.4

### 3.2 Latent profiles of psychological resilience

Four models were estimated during exploration. By comparing the information evaluation indicators and clinical significance, the AIC, BIC, and aBIC values of models 1–4 gradually decreased, the BLRT statistical values of models 3 and 4 were *p* < 0.05, and the entropy value of model 3 was greater than 0.9, indicating that the fitting effect of model 3 was good. Therefore, this study categorizes psychological resilience into 3 latent profiles (group 1, group 2, and group 3). Model fit statistics for all tested latent profile models are shown in **Table 2**.

**Table 2:** Fit statistics for latent profile analysis of resilience features and names of each latent profile.

Model	K	AIC	BIC	aBIC	Entropy	LMRT	BLRT
					*p*-value	*p*-value	
1	50	16,812.807	16,989.674	16,831.163			
2	76	15,287.181	15,556.018	15,315.082	0.867	0.134	0.132
3	102	14,730.213	15,989.216	14,767.659	0.939	0.0021^**^	0.0000^**^
4	128	14,620.241	15,073.019	146,67.232	0.820	0.6296	0.0000^**^

^**^p < 0.01.

**Figure 1:**
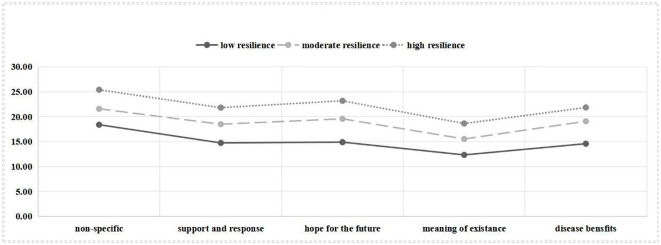
Exact mean of the three-profile mode of resilience.

The exact means of three latent profiles (profile 1, profile 2, and profile 3) on five dimensions of Resilience Scale Specific to Cancer (RS-SC) are shown in **Figure 1**. Each profile was named based on the scale’s means of five dimensions. Profile 1, which scored low on all five dimensions, is called the “Low Resilience” group. Profile 2, which scored moderately on each dimension, is called the “Moderate Resilience” group. Profile 3, which scored high on all five dimensions, is called the “High Resilience” group. The three profiles showed significant differences in average scores (*p* < 0.001) (see **Table 3**).

**Table 3:** Three resilience profiles’ exact mean in five dimensions of resilience scale (*N* = 254).

Variable	Low Resilience *n* = 45 (17.72%)		Moderate Resilience *n* = 113 (44.49%)		High Resilience *n* = 96 (37.80%)		*χ*^2^
	M	SE	M	SE	M	SE	
Non-specific resilience	18.38	2.84	21.57	3.02	25.39	2.92	112.165**
Disease benefits	14.58	2.51	19.07	1.74	21.83	2.21	145.129**
Support and response	14.73	2.31	18.48	1.96	21.80	1.78	160.002**
Hope for the future	14.89	2.75	19.57	2.25	23.18	1.85	159.618**
Meaning of existence	12.33	2.66	15.52	1.63	18.63	1.41	161.942**
Resilience	74.91	7.93	94.20	5.13	110.82	5.49	214.105**

**p < 0.01.

Profile 1 (“low resilience” group, 17.72%) showed the lowest means of all domains for resilience; among the domains, meaning of existence dimension had the lowest score, and the non-specific resilience score was relatively high. Profile 2 (“moderate resilience” group, 44.49%) showed moderate means of all domains but had several comparatively low means for meaning of existence. Profile 3 (“high resilience” group, 37.80%) showed the highest means of all domains for resilience. Of note, the 3 profiles were all lowly probable in meaning of existence.

### 3.3 Sociodemographic and disease characteristics of three latent profiles

The differences in the sociodemographic and disease factors of each profile are shown in **Table 4**. The chi-squared test revealed significant differences (*p* < 0.05) in gender, marital status, children, education level, annual income, insurance status, tumor site, time of diagnosis, history of tumor-related surgeries, initial recurrence, and treatment regimen among the patients in the different latent profiles.

**Table 4:** Socio-demographic and disease characteristics of three latent profiles.

Variable		Low Resilience *n* = 45 (17.72%)	Moderate Resilience *n* = 113 (44.49%)	High Resilience *n* = 96 (37.80%)	*χ*^2^	*p*
Gender	Female	25 (55.6%)	40 (35.4%)	29 (30.2%)	17.15	0.001**
	Male	20 (44.4%)	73 (64.6%)	67 (69.8%)		
Marital status	Unmarried	10 (22.2%)	20 (17.7%)	14 (14.6%)	108.488	0.001**
	Married	35 (77.8%)	93 (82.3%)	82 (85.4%)		
Children	No	12 (26.7%)	32 (28.3%)	20 (20.8%)	62.504	0.001**
	Yes	33 (73.3%)	81 (71.7%)	76 (79.2%)		
Education level	Middle school or lower	21 (46.7%)	46 (40.7%)	18 (18.8%)	48.906	0.001**
	High school	24 (53.3%)	54 (47.8%)	52 (54.2%)		
	College degree or higher	0 (0%)	13 (11.5%)	26 (27.1%)		
Employment status	Unemployed	31 (68.9%)	69 (61.1%)	25 (26.0%)	0.063	0.802
	Employed	14 (31.1%)	44 (38.9%)	71 (74.0%)		
Annual income (RMB)	<150,000	20 (44.4%)	39 (34.5%)	17 (17.7%)	8.984	0.011*
	150,000–300,000	10 (22.2%)	36 (31.6%)	25 (26.0%)		
	>300,000	15 (33.3%)	38 (33.6%)	54 (56.3%)		
Insurance status	No	19 (42.2%)	43 (38.1%)	19 (19.8%)	33.323	0.001**
	Yes	26 (57.8%)	70 (61.9%)	77 (80.2%)		
Tumor stage	I and II	23 (51.1%)	37 (32.7%)	43 (44.8%)	9.071	0.003**
	III and IV	22 (48.9%)	76 (67.3%)	53 (55.2%)		
Tumor type	Nasopharyngeal	21 (46.7%)	52 (46.0%)	40 (41.7%)	66.598	0.001**
	Oral cavity	9 (20.0%)	15 (13.3%)	18 (18.8%)		
	Larynx	8 (17.8%)	11 (9.7%)	9 (9.4%)		
	Oropharynx	7 (15.6%)	35 (31.0%)	29 (30.2%)		
Time of diagnosis	0–3 months	21 (46.7%)	39 (34.5%)	19 (19.8%)	7.000	0.030*
	3–6 months	11 (24.2%)	35 (31.0%)	25 (26.0%)		
	>6 months	13 (28.9%)	39 (34.5%)	52 (54.2%)		
History of tumor-related surgeries	No	17 (37.8%)	26 (23.0%)	25 (26.0%)	54.819	0.001**
	Yes	28 (62.2%)	87 (77.0%)	71 (74.0%)		
Initial recurrence	No	31 (68.9%)	77 (68.8%)	60 (62.5%)	27.229	0.001**
	Yes	14 (31.1%)	35 (31.3%)	36 (37.5%)		
Treatment regimen	X-ray + heavy ion	8 (17.8%)	20 (17.7%)	26 (27.1%)	11.512	0.009**
	Protons + heavy ion	15 (33.3%)	26 (23.0%)	21 (21.9%)		
	Protons	10 (22.2%)	44 (38.9%)	32 (33.3%)		
	Heavy ion	12 (26.7%)	23 (20.4%)	17 (17.7%)		

^*^p < 0.05, ^**^p < 0.01.

### 3.4 Predictors of three latent profiles

Our multiple logistic regression analysis revealed significant associations with psychological resilience. Female patients (*p* < 0.01), those with an education level of middle school or lower (*p* < 0.01), and those undergoing proton therapy (*p* < 0.01) demonstrated lower levels of resilience. Conversely, patients with a high school education level (*p* < 0.05), tumor staging at stage I and II (*p* < 0.01), no recurrence (*p* < 0.05), and undergoing X-ray + heavy ion therapy(*p* < 0.01) exhibited higher levels of resilience. The results are shown in **Table 5**.

**Table 5:** Multivariate logistic analysis of resilience latent profile (*N* = 254).

Group	Variables	*B*	SE	Wald *χ*^2^	*p*	OR	95% CI	
							Lower	Upper
Low Resilience	Intercept	−21.02	0.83	644.47	0.001^**^			
	Female	1.10	0.44	6.20	0.01^**^	3.01	1.27	7.17
	Unmarried	0.45	0.85	0.29	0.59	1.58	0.30	8.25
	No children	0.46	0.76	0.36	0.55	1.58	0.35	7.04
	Middle school or lower	20.12	0.47	1812.59	0.001^**^	549329541.30	217512879.50	1387333687.00
	High school	19.33	0.00	.	.	247355263.40	247355263.40	247355263.40
	Annual income: <150,000	1.46	2.27	0.41	0.52	4.30	0.05	370.74
	Annual income: 150,000–300,000	−2.27	1.50	2.29	0.13	0.10	0.01	1.96
	No insurance	−2.97	1.69	3.07	0.08	0.05	0.00	1.42
	Nasopharyngeal	0.84	0.66	1.61	0.20	2.32	0.63	8.53
	Oral cavity	0.12	0.71	0.03	0.86	1.13	0.28	4.55
	Larynx	1.44	0.75	3.64	0.06	4.22	0.96	18.51
	Stage I and II	0.32	0.83	0.15	0.70	1.38	0.27	6.96
	Time of diagnosis: 0–3 months	2.42	1.69	2.06	0.15	11.25	0.41	307.35
	Time of diagnosis: 3–6 months	2.47	1.54	2.58	0.11	11.85	0.58	241.85
	No surgery	0.10	0.85	0.01	0.91	1.11	0.21	5.84
	No recurrence	0.05	0.65	0.01	0.94	1.05	0.29	3.78
	X-ray + heavy ion	−1.59	0.83	3.66	0.06	0.20	0.04	1.04
	Protons + heavy ion	−0.19	0.70	0.07	0.79	0.83	0.21	3.28
	Protons	−1.75	0.69	6.32	0.01^*^	0.18	0.05	0.68
High Resilience	intercept	0.82	0.61	1.81	0.03^*^			
	Female	−0.12	0.34	0.11	0.74	0.89	0.46	1.74
	Unmarried	0.14	0.62	0.05	0.82	1.16	0.34	3.90
	No children	−0.67	0.54	1.56	0.21	0.51	0.18	1.47
	Middle school or lower	−0.67	0.44	2.32	0.13	0.51	0.22	1.21
	High school	−1.53	0.51	9.03	0.00**	0.22	0.08	0.59
	Annual income: <150,000	−3.22	2.11	2.32	0.13	0.04	0.00	2.51
	Annual income: 150,000–300,000	−1.88	1.37	1.90	0.17	0.15	0.01	2.21
	No insurance	0.12	0.96	0.02	0.90	1.12	0.17	7.35
	Nasopharyngeal	−0.28	0.44	0.41	0.52	0.76	0.32	1.79
	Oral cavity	0.77	0.50	2.37	0.12	2.17	0.81	5.81
	Larynx	0.17	0.59	0.08	0.77	1.19	0.38	3.75
	Stage I and II	1.09	0.55	3.92	0.05^*^	2.99	1.01	8.83
	Time of diagnosis: 0–3 months	2.10	1.91	1.22	0.27	8.20	0.20	343.40
	Time of diagnosis: 3–6 months	1.35	1.40	0.94	0.33	3.87	0.25	60.01
	No surgery	0.25	0.62	0.16	0.69	1.28	0.38	4.34
	No recurrence	−0.95	0.42	5.13	0.02^*^	0.39	0.17	0.88
	X-ray + heavy ion	1.35	0.60	5.03	0.03^*^	3.84	1.19	12.45
	Protons + heavy ion	0.38	0.54	0.49	0.49	1.45	0.51	4.16
	Protons	0.39	0.47	0.68	0.41	1.47	0.59	3.66

^*^p < 0.05, ^**^p < 0.01.

### 3.5 Analysis of different resilience profiles in quality of life

In our analysis, we found significant differences in role function, social function, pain, and overall quality of life among three resilience profiles (*p* < 0.05) (refer to **Table 6**). Specifically, the “high resilience” group had significantly higher scores in role function (*χ*^2^ = 8.348, *p* = 0.015), social function (*χ*^2^ = 15.015, *p* < 0.001), and overall quality of life (*χ*^2^ = 26.395, *p* < 0.001) compared to the “low resilience” group and “moderate resilience” group. Additionally, the “high resilience” group had a significantly lower pain score compared to the “low resilience” group and “moderate resilience” group (*χ*^2^ = 12.078, *p* < 0.05).

**Table 6:** Analysis of different resilience profiles in quality of life (*N* = 254).

Variable	Low Resilience *n* = 45 (17.72%)	Moderate Resilience *n* = 113 (44.49%)	High Resilience *n* = 96 (37.80%)	*χ*^2^	*p*
Physical function	92.15 ± 15.19	93.81 ± 12.11	94.38 ± 10.87	0.322	0.851
Role function	90.00 ± 17.19	91.15 ± 16.07	96.01 ± 10.47	8.348	0.015^*^
Emotional function	90.00 ± 13.13	90.93 ± 13.06	94.62 ± 8.72	5.386	0.068
Cognitive function	94.07 ± 13.37	95.13 ± 9.88	95.49 ± 9.20	0.143	0.931
Social function	86.30 ± 17.51	84.96 ± 13.02	94.10 ± 13.02	15.015	0.001^**^
Fatigue	15.53 ± 11.11	14.69 ± 11.50	13.13 ± 9.03	1.217	0.544
Nausea	4.20 ± 1.11	0.44 ± 2.69	0.35 ± 2.41	2.238	0.327
Pain	14.0 ± 17.41	11.59 ± 6.05	6.34 ± 1.91	12.078	0.002^**^
Breathe	11.94 ± 2.69	9.51 ± 2.95	12.09 ± 2.43	0.989	0.610
Insomnia	17.15 ± 10.37	19.55 ± 10.62	16.49 ± 6.6	3.763	0.152
Appetite	10.59 ± 3.70	13.53 ± 5.01	10.67 ± 3.82	0.276	0.875
Constipate	9.59 ± 2.96	11.03 ± 4.13	10.29 ± 3.13	0.668	0.716
Diarrhea	6.95 ± 1.48	4.41 ± 0.59	0	3.993	0.136
Economic	23.02 ± 14.07	22.85 ± 12.68	23.18 ± 11.81	0.708	0.702
Overall quality of life	74.63 ± 20.18	78.20 ± 14.90	87.78 ± 12.18	26.395	0.001^**^

^*^p < 0.05, ^**^p < 0.01.

## 4 Discussion

Three different resilience profiles for HNC parents were found in this study. The three resilience profiles were named the “low resilience” group, “moderate resilience” group, and “high resilience” group. The results showed that 44.49% (*n* = 113) of the patients belonged to the “moderate resilience” group, accounting for the majority of patients, which is consistent with a previous study on resilience in HNC patients (10). In other words, most HNC patients are able to successfully cope with the diagnosis of a tumor and its treatment, having moderate psychological resilience. However, it is worth noting that 17.72% (*n* = 45) of the patients belonged to the “low resilience” group, and their average resilience score was the lowest. Our study highlights the urgent need to identify and develop resilience-based therapies for HNC patients who fall into the “low resilience” group.

It is noteworthy that all three profiles exhibited low scores on the subscale of meaning of existence. Compared to prior literature, our findings align with the study conducted by Li et al. (16), which also reported low scores on the subscale of meaning of existence among HNC patients. These findings suggest that regardless of patients’ resilience profile, they face significant challenges in finding purpose and meaning in their lives. The absence of meaning may contribute to decreased role function, social function, and overall quality of life. Interventions such as counseling, support groups, and mindfulness-based techniques can all be effective in promoting meaning of existence and positivity among cancer patients (17).

### 4.1 Sociodemographic and disease characteristics of patients in different resilience profiles

This study found that gender, education level, tumor stage, initial recurrence, and treatment regimen are important factors that affect the resilience profile of patients. The findings of this study align with previous research that has explored the factors influencing resilience in cancer patients (18). Several studies have identified gender as a significant factor, although the specific impact may vary across different populations and cultural contexts (16, 19). For example, a study by Smith et al. (20) found that female cancer patients exhibited higher levels of resilience compared to male patients, while another study reported no significant gender differences (11). These inconsistencies highlight the need for further research to better understand the role of gender in resilience among cancer patients.

Similarly, the association between education level and resilience has been consistently reported in the literature. A study by Zahid et al. (18) found that higher education levels were associated with increased resilience in cancer patients. This suggests that education provides individuals with better coping strategies and resources to navigate the challenges of cancer treatment. Future interventions should focus on promoting health literacy and providing educational materials tailored to the specific needs of cancer patients.

The influence of tumor stage on resilience has also been widely studied. Consistent with our findings, a study demonstrated that advanced tumor stages were associated with lower levels of resilience in cancer patients undergoing radiotherapy (21). These findings emphasize the importance of providing targeted support to patients facing more advanced stages of cancer. Interventions should focus on addressing the unique physical and emotional challenges associated with advanced disease, such as pain management and supportive counseling services.

The impact of initial recurrence on resilience has been explored in several studies as well. Similar to our findings, a study by Lai et al. (22) reported that cancer patients who experienced a recurrence had lower levels of resilience compared to those who did not. This highlights the need for interventions that specifically address the emotional distress and uncertainty that accompany recurrence. Support systems should be in place to help patients cope with the fear of recurrence, provide psychological support, and assist in developing strategies for managing anxiety and stress.

The study also found that patients receiving proton therapy had lower psychological resilience levels compared to those undergoing X-ray and heavy ion therapy. This could be due to factors such as the physical characteristics of proton therapy (23), the psychological impact of the treatment, and the availability and effectiveness of supportive care. However, more research is needed to understand the underlying mechanisms and develop strategies to enhance resilience in patients undergoing proton therapy.

### 4.2 The quality of life of patients in different resilience profiles

This study investigated the quality of life of patients with HNC during radiotherapy. By analyzing the quality of life of patients with different resilience latent profiles, this study found that patients in the “low resilience” group had poorer role function and social function, more severe pain symptoms, and poorer overall quality of life. These findings highlight the significant impact of psychological resilience on various aspects of patients’ wellbeing and functioning.

The association between psychological resilience and role function is consistent with previous research (24). Resilience plays a crucial role in helping individuals adapt and maintain their roles and responsibilities, even in the face of adversity such as a cancer diagnosis. Patients with low resilience may experience difficulties in fulfilling their roles and responsibilities, which can negatively affect their overall functioning and wellbeing.

The impact of psychological resilience on social function is also noteworthy. Resilience enables individuals to maintain and develop social connections, seek support, and engage in meaningful social activities (25). Patients with low resilience may face challenges in maintaining social relationships and participating in social activities, leading to poorer social function and potential feelings of isolation.

The finding that patients with low resilience experience more severe pain symptoms aligns with the existing literature (26). A few studies have demonstrated a strong association between psychological resilience and pain perception (27). Patients with higher levels of resilience tend to exhibit better pain management strategies, increased pain tolerance, and improved overall wellbeing compared to those with lower resilience. These findings highlight the importance of addressing psychological resilience in the context of pain management. By implementing interventions that enhance resilience, healthcare providers can potentially help patients better cope with pain and improve their overall treatment outcomes.

Further research is needed to explore the underlying mechanisms linking psychological resilience and these outcomes, as well as to develop and evaluate effective interventions to enhance resilience in cancer patients. By doing so, healthcare providers can better support patients in managing the challenges associated with cancer and improve their overall wellbeing.

### 4.3 Limitations

A potential limitation of the current study is that we used a convenience sample of patients in a single region; thus, the scope of the study is relatively limited. Furthermore, this study only analyzed the predictive factors of the psychological resilience latent profile group of HNC patients using sociodemographic and disease factors. In the future, researchers need to explore the psychological quality factors of patients’ psychological resilience based on scientific theories to provide a theoretical basis for formulating intervention programs to improve patients’ resilience.

### 4.4 Clinical implications

According to the findings of this study, clinical nurses and researchers should focus on HNC patients who have a low quality of life due to a lack of psychological resilience. HNC patients with lower levels of resilience have poorer role function, social function, and overall quality of life and have more obvious pain symptoms. Therefore, it is very important to identify HNC patients with different levels of psychological resilience. This study also found that the level of psychological resilience of HNC patients can be divided into three latent profiles and that gender, education level, treatment regimen, tumor stage, and initial recurrence are important factors of the resilience latent profile. Although this study has limitations, it provides important data analysis and results about predictors of resilience in HNC patients and may be useful for future research and clinical interventions for patients.

## 5 Conclusion

The psychological resilience of HNC patients can be divided into three profiles: the “low resilience” group, the “moderate resilience” group, and the “high resilience” group. It is interesting to note that those who are female and have a low education level tend to have lower psychological resilience. This highlights the influence of socioeconomic and disease factors on resilience levels. Furthermore, HNC patients with lower resilience not only have poorer role function and social function, but also experience a lower overall quality of life, along with more severe pain symptoms. These findings underscore the importance of psychological resilience for HNC patients. By identifying patients with lower resilience, healthcare professionals can provide targeted interventions to improve their role and social wellbeing, overall quality of life, and pain management.

## Data availability statement

The original contributions presented in the study are included in the article/**Supplementary Material**. Further inquiries can be directed to the corresponding author.

## Ethics statement

The studies involving humans were approved by Shanghai Proton and Heavy ion Hospital. The studies were conducted in accordance with the local legislation and institutional requirements. The participants provided their written informed consent to participate in this study. Written informed consent was obtained from the individual(s) for the publication of any potentially identifiable images or data included in this article.

## Author contributions

LX: Data curation, Investigation, Methodology, Software, Writing – original draft, Writing – review & editing. HW: Conceptualization, Funding acquisition, Resources, Supervision, Visualization, Writing – review & editing. YZ: Conceptualization, Formal analysis, Project administration, Supervision, Validation, Visualization, Writing – review & editing. SW: Data curation, Investigation, Formal analysis, Writing – review & editing. MZ: Data curation, Investigation, Formal analysis, Writing – review & editing.
